# Estimating Ebola Treatment Needs, United States

**DOI:** 10.3201/eid2107.150286

**Published:** 2015-07

**Authors:** Gabriel Rainisch, Jason Asher, Dylan George, Matt Clay, Theresa L. Smith, Christine Kosmos, Manjunath Shankar, Michael L. Washington, Manoj Gambhir, Charisma Atkins, Richard Hatchett, Tim Lant, Martin I. Meltzer

**Affiliations:** Centers for Disease Control and Prevention, Atlanta, Georgia, USA (G. Rainisch, T.L. Smith, K. Cosmos, M. Shankar, M. Washington, C. Atkins, M.I. Meltzer);; Leidos, Reston, Virginia, USA (J. Asher, M. Clay);; Biomedical Advanced Research and Development Authority, Washington, DC, USA (D. George, R. Hatchett, T. Lant);; Monash University, Melbourne, Victoria, Australia (M. Gambhir)

**Keywords:** Ebola virus infection, hemorrhagic fever, Ebola virus disease, Ebola virus, viruses, epidemics, statistical models, models, hospital bed capacity, beds for Ebola disease, BED, hospital units, hospitalization, length of stay, communicable diseases

**To the Editor:** By December 31, 2014, the Ebola epidemic in West Africa had resulted in treatment of 10 Ebola case-patients in the United States; a maximum of 4 patients received treatment at any one time (*1*). Four of these 10 persons became clinically ill in the United States (2 infected outside the United States and 2 infected in the United States), and 6 were clinically ill persons medically evacuated from West Africa (Technical Appendix 1 Table 6).

 To plan for possible future cases in the United States, policy makers requested we produce a tool to estimate future numbers of Ebola case-patients needing treatment at any one time in the United States. Gomes et al. previously estimated the potential size of outbreaks in the United States and other countries for 2 different dates in September 2014 (*2*). Another study considered the overall risk for exportation of Ebola from West Africa but did not estimate the number of potential cases in the United States at any one time (*3*).

We provide for practicing public health officials a spreadsheet-based tool, Beds for Ebola Disease (BED) (Technical Appendix 2) that can be used to estimate the number of Ebola patients expected to be treated simultaneously in the United States at any point in time. Users of BED can update estimates for changing conditions and improved quality of input data, such as incidence of disease. The BED tool extends the work of prior studies by dividing persons arriving from Liberia, Sierra Leone, and Guinea into the following 3 categories: 1) travelers who are not health care workers (HCWs), 2) HCWs, and 3) medical evacuees. This categorization helps public health officials assess the potential risk for Ebola virus infection in individual travelers and the subsequent need for post-arrival monitoring (*4*).

We used the BED tool to calculate the estimated number of Ebola cases at any one time in the United States by multiplying the rate of new infections in the United States by length of stay (LOS) in hospital (Table). The rate of new infections is the sum of the rate of infected persons in the 3 listed categories who enter the United States from Liberia, Sierra Leone, or Guinea. For the first 2 categories of travelers, low and high estimates of Ebola-infected persons arriving in the United States are calculated by using low and high estimates of both the incidence of disease in the 3 countries and the number of arrivals per month (Table). Calculating the incidence among arriving HCWs required estimating the number of HCWs treating Ebola patients in West Africa (Technical Appendix 1, Tables 2–4). For medical evacuations of persons already ill from Ebola, we calculated low and high estimates using unpublished data of such evacuations through the end of December 2014.

**Table:** Calculated monthly rates of Ebola disease among persons arriving in the United States and additional secondary cases, 2014

Arriving persons		Input 1: infections/mo*	Input 2: at-risk population	Input 3: US arrival rate/mo†		Output 1: importations/mo‡	Output 4: additional secondary cases§	Output 2: total cases/mo‡
Non-HCW	Low	1	10,000	2,000		0.2	0	0.2
	High	3	10,000	3,000		0.9	2	2.7
HCW	Low	1	100	30		0.3	0	0.3
	High	5	100	60		3.0	2	9.0
Medical evacuations¶	Low	NA	NA	1		1.0	0	1
	High	NA	NA	3		3.0	0	3

*Infections in travelers who are not HCWs were based on the monthly incidence identified in World Health Organization situation reports during June–October 2014 (online Technical Appendix 1 Table 1) (*5*). The high value was the highest monthly incidence [September] rounded to the nearest whole number; the low value was set at 30% of the high value. Infections in HCWs were based on estimates of the number of HCWs in West Africa with and without Ebola virus infection at different times in the epidemic [online Technical Appendix 1 and Appendix 1 Tables 2–4]. HCW, health care worker; NA, not applicable. †The low estimate of US arrival rates for travelers who are not HCWs and both the low and high rates for HCWs were based on the count of screened airline passengers originating in Liberia, Sierra Leone, and Guinea in the month from mid-October through mid-November 2014 (Centers for Disease Control and Prevention [CDC], unpub. data). For the high US arrival rate for travelers who are not HCWs, we assumed a 50% increase over the low value [3,000 = 2,000 × 1.5] to approximate the arrival rate in 2013, before the epidemic (*3*). Rates of HCW arrivals were based on travelers who identified themselves as having worked in a health care facility during the previous 21 d during screenings at their airport of entry to the United States during November 5–December 1, 2014, and the exposure risk category assigned to them according to CDC’s Interim US Guidance for Monitoring and Movement of Persons with Potential Ebola Virus Exposure (*4,6*). The low estimate value of arrivals of HCWs (30 arriving HCWs) was approximately the lowest rate of high-risk and some-risk HCWs entering the United States. The high estimate value (60 arriving HCWs) was approximately the highest rate of high-, some-, and low-risk HCWs entering the United States (CDC, unpub. data). ‡Output 1 = (Input 1 / Input 2) × Input 3; Output 2 = Output 1 + (Output 1 × Input 4). See online Technical Appendix 1 for further details. §Assumed number of additional secondary transmissions occurring in the United States per primary case based on the range of experience from the outbreak: 1 imported case to the United States resulted in 2 secondary infections, and several case-patients have been treated without any secondary infections (*7*). ¶Number of medical evacuations was obtained from unpublished Medical Evacuation Missions Reports (US Department of Health and Human Services, unpub. data).

Although only 1 Ebola case has caused additional cases in the United States (*7*), we included the possibility that each Ebola case-patient who traveled into the United States would cause either 0 secondary cases (low estimate) or 2 secondary cases (high estimate) (Table). Such transmission might occur before a clinically ill traveler is hospitalized or between a patient and HCWs treating the patient (*7*). To account for the possibility that infected travelers may arrive in clusters, we assumed that persons requiring treatment would be distributed according to a Poisson probability distribution. Using this distribution enables us to calculate, using the BED tool, 95% CIs around the average estimate of arriving case-patients. The treatment length used in both the low and high estimate calculations was 14.8 days, calculated as a weighted average of the LOS of hospitalized case-patients treated in West Africa through September 2014 (Technical Appendix 1 Table 5) (*8*). We conducted a sensitivity analysis using LOS and reduced case-fatality rate of patients treated in the United States (Technical Appendix 1 Table 6).

For late 2014, the low estimate of the average number of beds needed to treat patients with Ebola at any point in time was 1 (95% CI 0–3). The high estimate was 7 (95% CI 2–13).

In late 2014, the United States had to plan and prepare to treat additional Ebola case-patients. By mid-January 2015, the capacity of Ebola treatment centers in the United States (49 hospitals with 71 total beds [*9*]) was sufficient to care for our highest estimated number of Ebola patients. Policymakers already have used the BED model to evaluate responses to the risk for arrival of Ebola virus–infected travelers, and it can be used in future infectious disease outbreaks of international origin to plan for persons requiring treatment within the United States.

Technical Appendix 1 Data inputs and assumptions; sensitivity analysis (length of stay and case-fatality rate); comparison with other published estimates; and limitations.

Technical Appendix 2 Beds for Ebola Disease (BED) model.

## References

[1] Ashkenas J, Buchanan L, Burgess J, Fairfield H, Grady D, Keller J, How many Ebola patients have been treated outside of Africa?: New York Times; 01/26/2015 [cited 2015 Feb 13]. http://www.nytimes.com/interactive/2014/07/31/world/africa/ebola-virus-outbreak-qa.html

[2] Gomes MFC, Piontti AP, Rossi L, Chao D, Longini I, Halloran ME, Assessing the international spreading risk associated with the 2014 West African Ebola outbreak. PLoS Curr. 2014;6. pii: ecurrents.outbreaks.cd818f63d40e24aef769dda7df9e0da5. 10.1371/currents.outbreaks.cd818f63d40e24aef769dda7df9e0da5 PMC4169359 25642360

[3] Bogoch II, Creatore MI, Cetron MS, Brownstein JS, Pesik N, Miniota J, Assessment of the potential for international dissemination of Ebola virus via commercial air travel during the 2014 west African outbreak. Lancet. 2015;385:29–35 . 10.1016/S0140-6736(14)61828-6 25458732 PMC4286618

[4] Brown CM, Aranas AE, Benenson GA, Brunette G, Cetron M, Chen TH, Airport exit and entry screening for Ebola—August–November 10, 2014. MMWR Morb Mortal Wkly Rep. 2014;63:1163–7 . 25503920 PMC4584540

[5] World Health Organization. Global Alert and Response (GAR). Situation reports with epidemiological data: archive. Situation report update—October 22, 2014. Ebola response roadmap situation report [cited 2014 Dec 24 ]. http://www.who.int/csr/disease/ebola/situation-reports/archive/en/

[6] Centers for Disease Control and Prevention. Interim US guidance for monitoring and movement of persons with potential Ebola virus exposure. December 24, 2014 [cited 2014 Dec 24]. http://www.cdc.gov/vhf/ebola/exposure/monitoring-and-movement-of-persons-with-exposure.html

[7] Chevalier MS, Chung W, Smith J, Weil LM, Hughes SM, Joyner SN, Ebola virus disease cluster in the United States—Dallas County, Texas, 2014. MMWR Morb Mortal Wkly Rep. 2014;63:1087–8 . 25412069 PMC5779510

[8] World Health Organization Ebola Response Team. Ebola virus disease in West Africa—the first 9 months of the epidemic and forward projections. N Engl J Med. 2014;371:1481–95. Epub 2014 Sep 22. 10.1056/NEJMoa1411100 25244186 PMC4235004

[9] Centers for Disease Control and Prevention. Current Ebola treatment centers. 12/31/2014 [cited 2014 Jan 5]. http://www.cdc.gov/vhf/ebola/healthcare-us/preparing/current-treatment-centers.html

