# Supplementary material for: Estimating Ebola Treatment Needs, United States
Source: Emerg Infect Dis. 2015 Jul;21(7):1273–5. doi: 10.3201/eid2107.150286 (PMC4816331; doi:10.3201/eid2107.150286)
Supplement: Technical Appendix 1 — Data inputs and assumptions; sensitivity analysis (length of stay and case-fatality rate); comparison with other published estimates; and limitations. [file 15-0286-Techapp-s1.pdf]

Article DOI: http://dx.doi.org/10.3201/eid2107.150286

Estimating Ebola Treatment Needs, United States

Technical Appendix

Data Inputs and Assumptions

General Travelers

This category consists of travelers who originate their travels to the United States from Liberia, Sierra Leone, or Guinea and who do not fall within the “health care worker” (HCW) or “medical evacuee” categories. The monthly number of travelers with Ebola entering the United States who are not health care workers (main text Table: Input 1) was based on the 1-month average incidence of Ebola per 10,000 population of the combined populations of Liberia, Sierra Leone, and Guinea (Technical Appendix 1 Table 1). The high estimate of the number of travelers arriving infected with Ebola virus (3 infections/10,000 persons at risk) is based on the highest monthly incidence (September) and the assumption that travelers have a risk for Ebola virus infection equal to that of the general population (*1*). This assumption is sometimes called “homogenous mixing.” A low estimate was calculated (1 infection/10,000 persons at risk) by assuming that most travelers are from a higher socioeconomic status, which enables them to live in conditions that may reduce their risk of being infected with Ebola virus and that exit screening might reduce the numbers of exposed or ill travelers. This lower risk was assumed to be 30% of that of the general population.

The arrival rate of travelers who are not HCWs (main text Table: Input 3) was based on the number of travelers currently reported as arriving in the United States whose inbound travel originated in Liberia, Sierra Leone, or Guinea. The low estimate for the arrival rate of travelers who are not HCWs was the arrival rate at the time of this analysis (≈2,000 arrivals/month [Centers for Disease Control and Prevention, unpub. data]), and the high estimate (3,000 arrivals/month) was chosen by assuming the arrival rate returns to preepidemic levels (a 50% increase in monthly arrivals from the arrival rate used in the low estimate).

HCWs

We defined an HCW as a person who has worked in ≥1 of the 3 West African countries in a capacity related to providing care to Ebola patients. The monthly rate of new HCW infections (main text, Table: Input 3) in West Africa was calculated by dividing the monthly number of reported Ebola cases in HCWs (at different time points in the epidemic) by estimates of the total HCW population exposed as a result of staffing Ebola Treatment Units (ETUs) (Technical Appendix 1 Table 4) (*1*). A lower estimate of the rate of infected HCWs in West Africa was calculated by using the 3-month average number of cases reported among HCWs at the midpoint of the outbreak (36/month, calculated July 2014) (Technical Appendix 1 Table 3) and the highest estimate of HCWs in the 3 West African countries (4,172 workers/1000 ETU beds) (Technical Appendix 1 Table 4). This number of HCWs assumes that all HCWs, regardless of their type of employment, are at higher risk than the general population for exposure to Ebola (Technical Appendix 1 Table 2). A higher estimate of the rate of infections among HCWs was calculated by using the maximum 3-month average infections among HCWs to date (129/month, calculated in October 2014) (Technical Appendix 1 Table 3), and the lowest HCW population at risk (2,677 workers/1,000 ETU beds) (Technical Appendix Table 2). This number of HCWs assumes that a smaller subset of staff, based on their position (i.e., those more likely to have patient encounters), are at higher risk for Ebola infection.

The arrival rate of HCWs to the United States was based on 1) the number of travelers who identified themselves as having worked in a health care facility during the previous 21 days and 2) the risk category ("high," "some," or "low") assigned to them during enhanced entry screening at their airport of entry to the United States during November 5–December 1, 2014 (*2*,*3*). The low estimate value of arrivals of HCWs (30 arrivals/month) was approximately the lowest rate of "high-" and "some-risk" HCWs entering the United States (main text Table: Input 3) during the timeframe examined. The high estimate value (60 arrivals/month) was approximately the highest rate of high-, some-, and low-risk HCWs entering the United States.

Medical Evacuees

This category comprises persons who already have symptomatic Ebola-related illness and who are consequently flown to the United States for treatment in special aircraft with a special containment apparatus. Patients in this category may include HCWs who are already clinically ill with Ebola. Patients in this category do not include persons who have had a "high-risk" exposure

in an affected country who enter the United States without clinical symptoms: Such persons do not require an ETU bed upon arrival but they may be admitted if they receive investigational therapies, such as postexposure prophylaxis. Based on the 3-month experience during the outbreak during August–October 2014, the number of medical evacuees to the United States was assumed to be either 3 or 1 per month (main text Table: Input 3). The high estimate (3 persons) was chosen to match the observed monthly average of the number of evacuees from West Africa to all other countries in the world (including the United States).

Secondary Transmission

Secondary transmission may occur during the period in which a traveler is clinically ill but before he or she is placed in an isolated hospital bed. Some secondary transmission may also occur between the ill patient and the US-based HCWs treating the patient (*4*). The number of secondary transmissions per each HCW and non-HCW case imported to the United States was assumed to be either 0 (low estimate) or 2 (high estimate) (main text Table: Input 4). The high estimate (2 cases) was based on the number of secondary transmissions that occurred during treatment of the first case diagnosed in the United States (*4*). Since this cluster, no secondary transmissions have occurred in the United States and a number of additional public health and hospital preparedness measures (including updated guidance for HCW's use of personal protective equipment and widespread training efforts) have been put in place to reduce and potentially eliminate such risk. Thus, we assumed 0 secondary transmissions for the low estimate. For medical evacuations, it was assumed that no secondary transmissions (0) occur during treatment of this category of infected persons.

In-hospital length of stay (LOS) was calculated as a weighted average of the LOS among hospitalized case-patients treated in Africa through September 2014 (Technical Appendix 1 Table 5) (*5*). The weighting was based on the proportions of patients with Ebola who recovered and died during treatment. The LOS used was 18 days for survivors and 10 days for nonsurvivors. Combining these values with the observed 40% case-fatality rate (CFR) resulted in a weighted average LOS of 14.8 days (Technical Appendix 1 Table 5) (*5*).

Sensitivity Analysis: Length of Stay and Case-Fatality Rate

A sensitivity analysis of LOS was also conducted in which LOS were based on case-patients treated in the United States through November 2014. Although few in number (n = 10), case-patients treated in the United States could have longer average LOS of 22.4 days and improved survival of 80% (i.e., CFR 20%). Case-patients treated in West Africa had an average LOS of 14.8 days and CFR 40% cases treated in Africa (Technical Appendix 1 Table 6).

When data on LOS and survival were used from case-patients treated in the United States (in the sensitivity analysis) the low estimate was still 1, but the 95% CI widened slightly (95% CI 0–4). The high estimate increased from 7 cases to 12 cases (95% CI 5–19).

Comparison with Other Published Estimates

Our estimates are within the range of other published estimates (*6*,*7*). Using a similar, incidence-based risk calculation (based on incidence in September 2014), Bogoch et al. estimated, assuming unrestricted airline travel, 7.17 Ebola-infected non-HCW travelers per month from West Africa to all destinations (*6*). Gomes et al. estimated (in September 2014) a 25% probability of 7 US cases (range 2–14) occurring in December 2014 by using a spatial, stochastic, and individual-based epidemic model (*7*). This estimate matches our high estimate of 7 (95% CI 2–13).

Limitations

The findings in this report are subject to several limitations. First, this analysis does not account for the possibility of the outbreak worsening in the future. If the incidence increases among the general population or HCWs, so would the rate of importations if air travel arrival rates remained the same. If Ebola becomes established in other countries (particularly those with many travelers to the United States) the rate of importation may also increase. However, our BED tool can be used to update and reestimate the risk for imported cases of Ebola. Second, this analysis does not specifically evaluate the effect of travel restrictions, such as reductions in airline traffic and capacity, and exit screenings (which could decrease the risk for travel by symptomatic persons or persons with higher exposure risks). Imposing reductions in air travel

may not have a notable impact. Gomes et al. found that reducing air travel may delay importation only by a few weeks but not prevent or reduce the rate of importation (*7*). Again, our BED tool can be used to explore the potential impact of a decrease or increase in the number of monthly arrivals from West Africa. Third, we assumed that secondary cases will be very limited and easy to contain, thus preventing further infections (i.e., no tertiary cases will occur). Fourth, the upper limit for the number of non-HCW travelers with Ebola was calculated by assuming that these travelers have a risk for infection equal to that of the general population in the 3 primarily affected West African countries. Because most travelers are likely to have a higher socioeconomic status than persons in the general population, and consequently, a lower risk for Ebola infection, this assumption most likely overestimates the risk for infection among travelers.

As an alternative (as noted in Appendix Data Inputs and Assumptions, General Travelers) we estimated in the lower limit calculation, the impact of assuming that travelers had a level of risk that is one third that of the general population in the 3 affected countries. This reduction in risk for infection among travelers, compared with the general population, may still overestimate the actual risk. Again, the BED tool can be used to explore the impact of assuming a different level of reduction in risk (either higher or lower than what we assumed). Finally, these results may notably underestimate or overestimate the likelihood of HCWs entering the United States from West Africa who are infected with Ebola because data on this traveler category are insufficient. For instance, the number of HCWs working in West Africa and the number of Ebola patients being treated in non-ETU settings (e.g., hospitals, clinics) is unknown. As a result, this analysis calculated the risk for exposure to Ebola for HCWs from limited data on the number of HCWs in ETUs; and assumed this risk was equal for all HCWs, irrespective of the setting in which they worked. Recent evidence, however, indicates that HCWs in ETUs constitute <5% of all Ebola infections among HCWs (*8*). Furthermore, even if the risk to HCWs could be reliably calculated, it cannot be determined how it applies to workers entering the United States because the data on self-declared HCWs obtained from airport screenings do not include specific data fields that capture where HCWs worked and what they did in West Africa.

References

1. World Health Organization. Global Alert and Response (GAR). Situation reports with epidemiological data: archive. Situation report update—October 22, 2014. Ebola response roadmap situation report [cited 2014 Dec 24]. http://www.who.int/csr/disease/ebola/situation-reports/archive/en/

2. Brown CM, Aranas AE, Benenson GA, Brunette G, Cetron M, Chen TH, et al. Airport exit and entry screening for Ebola—August–November 10, 2014. MMWR Morb Mortal Wkly Rep. 2014;63:1163–7. PubMed

3. Centers for Disease Control and Prevention. Interim US guidance for monitoring and movement of persons with potential Ebola virus exposure. December 24, 2014 [cited 2014 Dec 24]. http://www.cdc.gov/vhf/ebola/exposure/monitoring-and-movement-of-persons-with-exposure.html

4. Chevalier MS, Chung W, Smith J, Weil LM, Hughes SM, Joyner SN, et al. Ebola virus disease cluster in the United States—Dallas County, Texas, 2014. MMWR Morb Mortal Wkly Rep. 2014;63:1087–8. PubMed

5. World Health Organization Ebola Response Team. Ebola virus disease in West Africa—the first 9 months of the epidemic and forward projections. N Engl J Med. 2014;371:1481–95. Epub 2014 Sep 22. PubMed http://dx.doi.org/10.1056/NEJMoa1411100

6. Bogoch II, Creatore MI, Cetron MS, Brownstein JS, Pesik N, Miniota J, et al. Assessment of the potential for international dissemination of Ebola virus via commercial air travel during the 2014 west African outbreak. Lancet. 2015;385:29–35. PubMed http://dx.doi.org/10.1016/S0140-6736(14)61828-6

7. Gomes MFC, Piontti AP, Rossi L, Chao D, Longini I, Halloran ME, et al. Assessing the international spreading risk associated with the 2014 West African Ebola outbreak. PLoS Curr. 2014;6: pii: ecurrents.outbreaks.cd818f63d40e24aef769dda7df9e0da5. PubMed

8. Matanock A, Arwady MA, Ayscue P, Forrester JD, Gaddis B, Hunter JC, et al. Ebola virus disease cases among health care workers not working in Ebola Treatment Units—Liberia, June–August, 2014. MMWR Morb Mortal Wkly Rep. 2014;63:1077–81. PubMed

9. US Census Bureau. International programs: international data base. US Department of Commerce; December 2013 [cited 2014 Nov 6]. http://www.census.gov/population/international/data/idb/informationGateway.php

Technical Appendix 1 Table 1. Monthly Incidence of Ebola among the general population, Liberia, Sierra Leone, and Guinea, 2014

Month date range	Monthly new cases in all 3 countries*	Incidence rate per 10,000†
May 29–Jun 29	320	0.16
Jun 29–Jul 29	615	0.31
Jul 29–Aug 29	1,229	0.61
Aug 29–Sep 29	6,195	3.10
Sep 29–Oct 29	4,890	2.45

*Infections obtained from World Health Organization situation reports (*1*).
†Calculated using a 3-country population of 20 million (*9*).

Technical Appendix 1 Table 2. Numbers of high- and low-risk HCWs, by personnel type, West Africa, 2014

Type of personnel*	No. national staff/1,000 beds†	No. international staff/1,000 beds†	Total
High-risk HCW			
Water and sanitation	125	38	163
Health practitioner	163	25	188
Physician	0	63	63
Nurse	400	75	475
Nurse aid	100	0	100
Hygienist	525	0	525
Sprayer	300	0	300
Laundry attendant	100	0	100
Laborer	675	0	675
Cleaner	50	0	50
Plumber	13	0	13
Laundry worker	25	0	25
Subtotal	2,476	201	2,677
Low-risk HCW			
Medical focal point	0	13	13
Logistician	0	13	13
County health officer	50	13	63
Epidemiologist	0	13	13
Administrator	0	13	13
Dispenser	13	0	13
Maternal health counselor	13	0	13
Waste manager	50	0	50
Watchmen	425	0	425
Laborer supervisor	25	0	25
Carpenter	125	0	125
Electrician	63	0	63
Cook	50	0	50
Supply staff	50	0	50
Generator assistant	13	0	13
Warehouse manager	25	0	25
Warehouse laborer	88	0	88
Coordinator	0	13	13
Medical coordinator	0	13	13
Log supply worker	0	13	13
Log coordinator	0	13	13
Driver	288	0	288
Radio operator	50	0	50
Bike rider	25	0	25
Mapper	25	0	25
Subtotal	1,378	117	1,495
Total: Low- and high-risk HCWs	3,854	318	4172

*Personnel types that had Ebola virus infections in Liberia during June and August were defined as being high-risk (*8*); all others were categorized as low-risk. HCW, health care worker.
†The number of HCWs by personnel type was obtained from unpublished reports from Ebola Treatment Units in Liberia, Sierra Leone, and Guinea (Centers for Disease Control and Prevention, unpub. data).

Technical Appendix 1 Table 3. Number of Ebola cases among HCWs, West Africa, 2014 *

Month	Liberia	Sierra Leone	Guinea	Total	3-mo average
Mar	0	0	0	0	NA
Apr	2	0	18	20	NA
May	0	0	1	1	7
Jun	8	31	1	40	20
Jul	51	14	1	66	36
Aug	86	8	24	118	75
Sep	41	52	22	115	100
Oct	117	22	15	154	129

*Data from World Health Organization Situation Reports (*1*). NA, not applicable.

Technical Appendix 1 Table 4. Rates of infection for HCWs, West Africa

Estimate	Input 1: no. HCW cases/month*	Input 2: no. HCWs exposed/1,000 ETU beds†	Input 3: no. ETU beds‡	Output: Rate of infection/100 HCWs§
Low	36	4,172	1,040	1
High	129	2,677	1,040	5

*The average number of new HCW infections in West Africa at the outbreak's midpoint (36 during May–July) was used to calculate the low rate of infection, and the average number of new HCW infections in the most recent 3 mo (129 during August–October) was used to calculate the high rate of infection (refer to Technical Appendix 1 Table 3.) ETU, Ebola Treatment Unit; HCW, health care worker.
†The number exposed was based on the type of HCWs working in ETUs (see Technical Appendix 1 Table 2). For the low estimate calculation, we considered all HCWs as exposed (i.e., the sum low- and high-risk personnel). The high estimate calculation was based on the high-risk personnel only, under the assumption that a smaller subset of staff, based on their position (i.e., those more likely to have patient encounters) are at higher risk for Ebola virus infection.
‡The total number of ETU beds at the end of October among the primarily affected countries of Liberia, Guinea, and Sierra Leone (Centers for Disease Control and Prevention, unpub. data).
§Output (rounded to the nearest whole number) = [(Input 1)/(Input 2/1000 × Input 3)] × 100.

Technical Appendix 1 Table 5. Epidemiology data inputs: calculating LOS in hospitals, West Africa and United States, 2014

	Value	
Patient group or health outcome	Treated in Africa*	Treated in United States†
Survivors, d	18	28
Nonsurvivors, d	10	10
CFR, %	40t	20
Weighted Average LOS‡	14.8	22.4

*Based on the average interval from hospitalization to discharge + 1 SD; for survivors this was 11.8 d (SD 6.1), and for nonsurvivors it was 4.2 d (SD 6.4) (*9*). CFR, case-fatality rate; LOS, length of stay.
†Survivors' LOS (during treatment at US hospitals only) (n = 8) was based on 19.4 d + 1 SD of 8.8. Nonsurvivors' LOS (during treatment in US hospitals only) (n = 2) was the maximum LOS from the observed range of 2–10 d. CFR was obtained from 2 deaths of 10 case-patients treated (see Technical Appendix 1 Table 6).
‡Weighted Average LOS = LOS for survivors × (1-CFR proportion) + LOS for nonsurvivors × CFR.

Technical Appendix 1 Table 6. Length of stay data for each of the 10 Ebola patients treated in the United States, August 21–November 17, 2014

Patient	Date admitted to US facility*	Outcome	Date of discharge or death*	Length of stay, d†
1	Aug 2[1]	Lived	Aug 21	19
2	Aug 5[2]	Lived	Aug 19[3]	14
3	Sep 4	Lived	Sep 25	21
4	Sep 9	Lived	Oct 16‡	38
5	Sep 28	Died	Oct 8	10
6	Oct 6	Lived	Oct 21	15
7	Oct 11	Lived	Oct 24	13
8	Oct 14	Lived	Oct 28[4]	14
9	Oct 23	Lived	Nov 11	20
10	Nov 15	Died	Nov 17	2

*Source is Wikipedia unless indicated otherwise (cited 2015 Feb 19).
http://en.wikipedia.org/wiki/Ebola_virus_cases_in_the_United_States:
1. http://www.cbc.ca/news/world/ebola-outbreak-u-s-missionary-nancy-writebol-leaves-liberia-tuesday-1.2726884
2. http://www.nbcnews.com/storyline/ebola-virus-outbreak/nancy-writebol-american-ebola-patient-arrives-u-s-n172706
3. https://news.yahoo.com/hospital-discuss-discharge-ebola-patients-100148319.html
4. http://www.cnn.com/2014/10/28/health/us-ebola/
†Calculated as difference between date of discharge or death and admit date columns in this table.
‡Date of a statement indicating that the patient "would be released in the near future."
